# Beam width and arm position but not cognitive task affect walking balance in older adults

**DOI:** 10.1038/s41598-022-10848-y

**Published:** 2022-04-27

**Authors:** Andréia Abud da Silva Costa, Tibor Hortobágyi, Rob den Otter, Andrew Sawers, Renato Moraes

**Affiliations:** 1 Ribeirão Preto Medical School, Graduate Program in Rehabilitation and Functional Performance, University of São Paulo, São Paulo, Brazil; 2 Biomechanics and Motor Control Lab, School of Physical Education and Sport of Ribeirão Preto, University of São Paulo, São Paulo, Brazil; 3 Center for Human Movement Sciences, University of Groningen Medical Center, Groningen, The Netherlands; 4 Somogy County Kaposi Mór Teaching Hospital, Kaposvár, Hungary; 5 Department of Sport Biology, Institute of Sport Sciences and Physical Education, University of Pécs, Pécs, Hungary; 6 Division of Training and Movement Sciences, Research Focus Cognition Sciences, University of Potsdam, Potsdam, Germany; 7 Department of Kinesiology, Hungarian University of Sports Science, Budapest, Hungary; 8 Department of Kinesiology, University of Illinois at Chicago, Chicago, IL USA; 9 University Medical Center Groningen, Antonius Deusinglaan 1, 9713 AV Groningen, The Netherlands

**Keywords:** Motor control, Neural ageing

## Abstract

Detection of changes in dynamic balance could help identify older adults at fall risk. Walking on a narrow beam with its width, cognitive load, and arm position manipulated could be an alternative to current tests. Therefore, we examined additive and interactive effects of beam width, cognitive task (CT), and arm position on dynamic balance during beam walking in older adults. Twenty older adults (69 ± 4y) walked on 6, 8, and 10-cm wide beams (2-cm high, 4-m-long), with and without CT, with three arm positions (free, crossed, akimbo). We determined cognitive errors, distance walked, step speed, root mean square (RMS) of center of mass (COM) displacement and trunk acceleration in the frontal plane. Beam width decrease progressively reduced distance walked and increased trunk acceleration RMS. Step speed decreased on the narrowest beam and with CT. Arm crossing decreased distance walked and step speed. COM displacement RMS and cognitive errors were not affected by any manipulation. In conclusion, distance walked indicated that beam width and arm position, but less so CT, affected dynamic balance, implying that beam walking has the potential to become a test of fall risk. Stability measurements suggested effective trunk adjustments to control COM position and keep dynamic balance during the task.

## Introduction

An assessment of postural control of dynamic balance could identify older adults susceptible to mobility disability and falls^1^. Functional tests indirectly evaluate dynamic balance through walking speed or clinical scales (e.g., Berg Balance Scale and Tinetti Assessment Tool) but fail to induce an actual loss of balance^2^. These tests are also limited by ceiling and/or floor effects, inconsistencies in detecting fallers, and a lack of sensitivity for intervention or disease evolution. In addition, complex tests such as the BESTest, take a long time to administer and are designed to assess abilities other than dynamic balance. Beam walking is an emerging test paradigm to assess dynamic balance^1, 3, 4^, the ability to keep the center of mass (COM) within the base of support (BoS) while walking. Reductions in BoS, as on a beam, increase the likelihood of the COM moving outside of BoS while it pivots over the stance leg during gait. Instability may result in a step-off, i.e., a “loss of balance”^1^. Beam walking performance, i.e., distance walked on the beam without stepping-off, directly addresses stability in a task-specific manner. Thus, the beam walking test does not rely on COM velocity as an outcome. Instead, it assesses how the nervous system controls fluctuations in COM acceleration induced by instabilities during walking. Such properties make beam walking highly specific to balance control during gait, focusing the test on control rather than walking speed as a gross outcome^1^. Manipulation of beam width, arm position, and the cognitive load can generate additive and interactive effects, further increasing task complexity and potentially enhancing its sensitivity and specificity. Compared with crude measures of walking speed by functional tests, beam walking could perhaps more effectively detect changes in dynamic balance in different populations, with short administration time, low cost, and set-up burden in clinics and retirement homes.

One way to increase the difficulty of beam walking and challenge dynamic balance is to change the width of the beam^3–5^. As beam width decreases so does the BoS, increasing the demand for postural control to maintain dynamic balance^3^. Indeed, narrow beams can challenge even healthy young adults’ dynamic balance and reduce the distance walked^6^. In healthy older adults, reductions in beam walking distance are greater independent of foot posture and stepping pattern (comfortable, heel-to-toe, tandem)^3^. Relative to functional tests, variation in beam width minimizes ceiling and floor effects and produces high specificity and sensitivity^7^, making this test ecologically attractive in geriatric and clinical settings.

Healthy aging reduces gait automation and increases cortical involvement^8^, especially fronto-cortical control^9^. Increasing motor task difficulty is also associated with increased cortical control in healthy aging^10^. Thus, this greater dependence on cognitive function in both tasks makes the cognitive resources sharing more difficult^9, 10^. The motor-cognitive interaction can be dramatic during beam walking, reducing performance in older adults to approximately 0.5 m of the 4 m maximum^10^. Considering the limited cognitive resources when facing more difficult motor tasks and that we perform cognitive and motor tasks concurrently, adding a cognitive task to beam walking has the potential to increase test diagnostic accuracy.

While the effects of beam width and dual-tasking on beam walking performance have been examined^3, 4, 10^, the role of the arm position has received less attention^5^. Ankle and hip strategies do not consider upper limb movements during postural tasks^11^; the arms do play a role in maintaining dynamic balance in response to perturbations^5, 11, 12^, as arms increase lateral stabilization during steady-state walking^12^ or while negotiating unexpected slips during walking^13^. Arm swing counterbalances the momentum produced by the legs around the vertical axis during walking, reducing vertical ground reaction moments and metabolic cost of transport^14^. The effects of arm position (free, crossed over the chest, and akimbo—placing hands on hips while elbows point outward) on beam walking performance have yet to be systematically examined^15, 16^. Limiting arm movements is likely to increase the difficulty in controlling balance, potentially providing an additional way to modify the challenge of beam walking and enhance its sensitivity, specificity, and ecological validity.

Taken together, the purpose of the present study was to determine additive and interactive effects of beam width, arm position, and cognitive task on dynamic balance during beam walking in older adults. We expected to find that mechanical (beam width), cognitive, and postural (arm position) demands would interactively and additively affect dynamic balance measured by beam walking performance metrics (distance, speed), trunk acceleration, and COM control.

## Results

### Sample characteristics

Participants (*n* = 20) were between 65 and 76 years old, healthy, physically active, and had unimpaired functional balance and cognition (Table 1). One participant reported one fall in the previous year.

**Table 1:** Mean and standard deviation (±) of the participants' physical, behavioral, and cognitive variables (*n* = 20).

Parameters	Values
Sex	6 M/14F
Age (years)	69 ± 4
Body Mass (kg)	68.6 ± 12.7
Height (m)	1.61 ± 0.07
Foot Width (cm)	9.9 ± 0.7
Education (years)	12 ± 4
IPAQ^a^ (METs.min^-1^)	3134.4 ± 2720.7
Mini-Mental State Exam (points)^b^	28.3 ± 1.2
Mini-BEST Test (points)^c^	27.4 ± 0.8
Trail Making Test – Part A (s)^d^	44.5 ± 9.6
Trail Making Test – Part B (s)^d^	89.9 ± 35.9
Digit-Symbol Substitution Test^e^	38.1 ± 15.9

^a^Cut-off points for physical activity level: high = at least 3.000 METs.min^-1^ per week, moderate = at least 600 METs.min^-1^ per week, and low = less than 600 METs.min^-1^ per week^42^.

^b^Scores close to 30 points (maximum punctuation) indicate the absence of cognitive deficit. Cut-off points based on time of education in a Brazilian population: No schooling = 20 points; 1–4 years of schooling = 25 points; 5–8 years of schooling = 26 points; 9–11 years of schooling = 28 points; More than 11 years of schooling = 29 points^35^. Twelve of the twenty participants scored in the respective cut-off for their education level or higher, while the others 8 scored a maximum of 2 points below the indicated.

^c^Scores close to 28 points (maximum punctuation) indicate a low risk for falls^36^.

^d^Score stratification is based on age and time of education^37^. None of the participants scored both parts of the test in the expected stratification.

^e^Number of correct responses completed in 90 seconds^38^.

### Beam width effect

**Figure 1:**
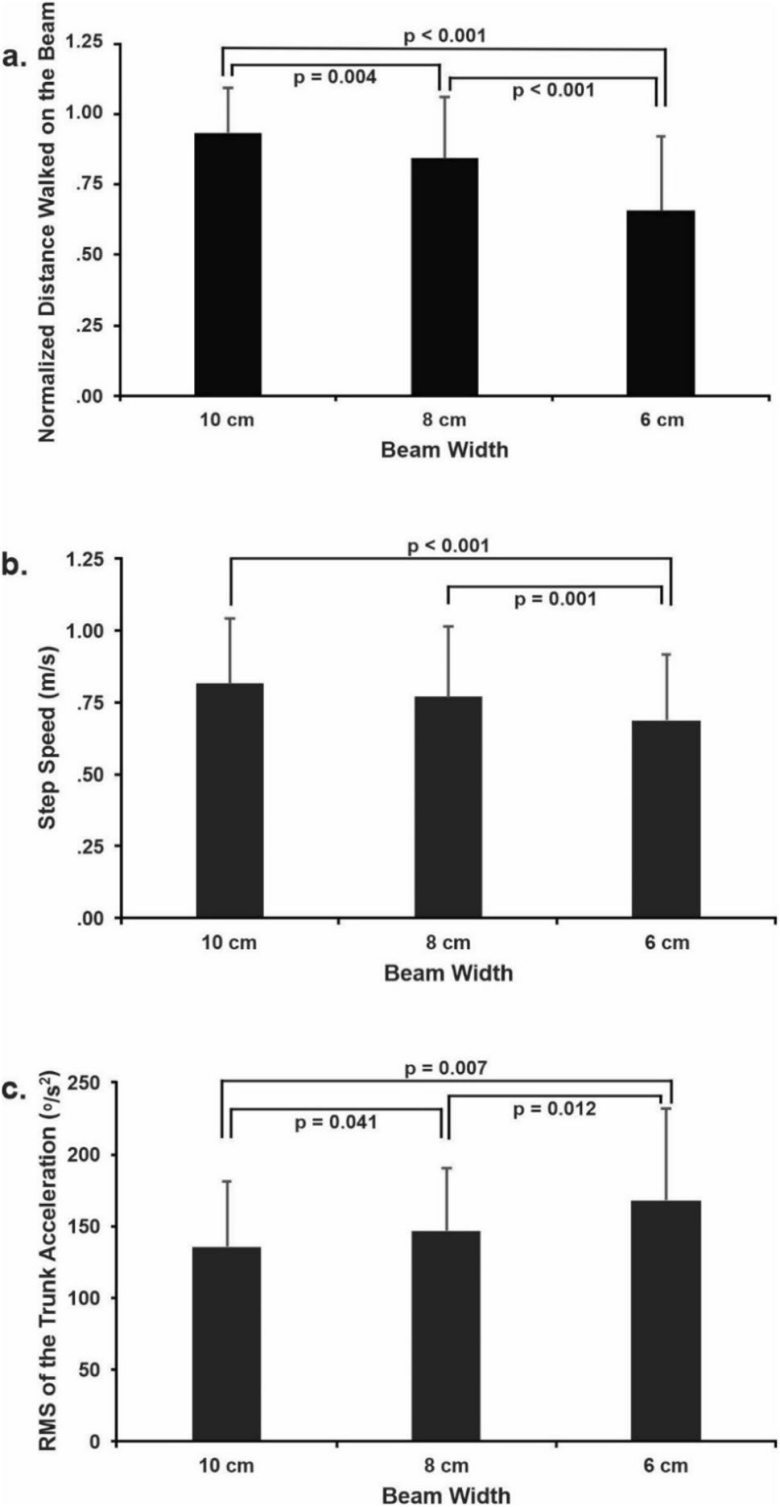
Mean and standard deviation of the beam width main effect for the normalized walking distance on the beam (**a**), step speed (**b**), and RMS of the trunk acceleration (**c**). The horizontal lines indicate pairwise differences with the respective *p*-value.

There was a main effect of beam width for normalized distance walked on the beams (F_2, 38_ = 40.285; *p* < 0.001; ${\eta }_{p}^{2}$=0.68), step speed (F_2,38_ = 18.539; *p* < 0.001; ${\eta }_{p}^{2}$=0.494), and trunk acceleration RMS (F_2, 38_ = 11.352; *p* = 0.002; ${\eta }_{p}^{2}$=0.374). Distance walked was 22.6% and 29.3% shorter on the 6-cm (0.65 ± 0.26) compared to the 8-cm (0.84 ± 0.21; *p* < 0.001; d = 0.97) and 10-cm (0.92 ± 0.16; *p* < 0.001; d = 1.66) wide beams, respectively. Distance walked was also 8.7% shorter on the 8-cm beam wide compared to the 10-cm beam wide (*p* = 0.004; d = 0.66, Fig. 1A). Step speed was 10.4% and 15.8% slower on the 6-cm (0.69 ± 0.23 m/s) than on 8-cm (0.77 ± 0.24 m/s, *p* = 0.001; d = 0.43) and 10-cm (0.82 ± 0.23 m/s, *p* < 0.001; d = 0.7) wide beams, respectively (Fig. 1B). The RMS of trunk acceleration in frontal plane was 14.7% and 24.0% higher on the 6-cm (168.12 ± 63.37 º/s^2^) than on the 8-cm (146.56 ± 43.96 º/s^2^; *p* = 0.012; d = 0.5) and the 10-cm (135.56 ± 45.77 º/s^2^; *p* = 0.007; d = 0.72) wide beams, respectively. RMS was also 8.1% higher on the 8-cm than on the 10-cm wide beam (*p* = 0.041; d = 0.3, Fig. 1C). No beam width effect was found for RMS of COM displacement or cognitive error (see Table 2 for means and standard deviations of all conditions).

**Table 2:** Mean and standard deviation (±) of the root mean square (RMS) of center of mass (COM) displacement and cognitive error for all beam width and arm position conditions.

	Cognitive task	Arm position	10-cm beam	8-cm beam		6-cm beam
RMS of COM displacement (m)	Without	Free	0.008 ± 0.003	0.007 ± 0.005		0.008 ± 0.004
		Akimbo	0.008 ± 0.003	0.009 ± 0.004		0.008 ± 0.003
		Crossed	0.01 ± 0.008	0.009 ± 0.003		0.007 ± 0.003
	With	Free	0.007 ± 0.003	0.008 ± 0.005		0.009 ± 0.004
		Akimbo	0.01 ± 0.006	0.01 ± 0.005		0.008 ± 0.003
		Crossed	0.01 ± 0.003	0.01 ± 0.005		0.009 ± 0.004
Cognitive error		Free	0.12 ± 0.16	0.19 ± 0.36		0.16 ± 0.26
		Akimbo	0.13 ± 0.17	0.09 ± 0.13		0.12 ± 0.23
		Crossed	0.15 ± 0.16	0.09 ± 0.14		0.06 ± 0.15

### Cognitive task effect

**Figure 2:**
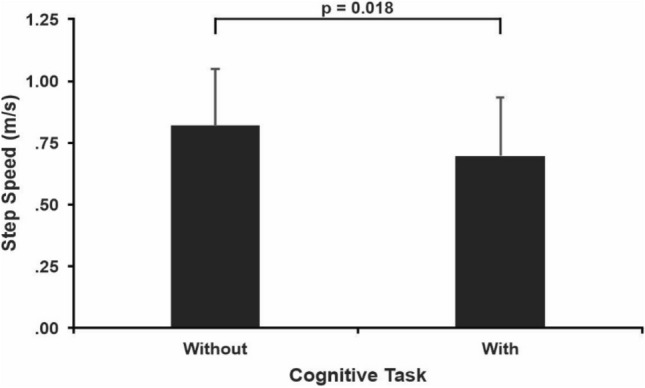
Mean and standard deviation of the cognitive task main effect for the step. The horizontal lines indicate pairwise differences with the respective p-value.

There was a main effect of cognitive task for step speed (F_1, 19_ = 6.751; *p* = 0.018; ${\eta }_{p}^{2}$=0.262). Step speed was 14.6% slower in the presence of the cognitive task (0.69 ± 0.24 m/s) than without it (0.82 ± 0.23 m/s; d = 0.6, Fig. 2). The ANOVA did not identify any effect of cognitive task on the RMS of COM displacement (Table 2).

### Arm position effect

**Figure 3:**
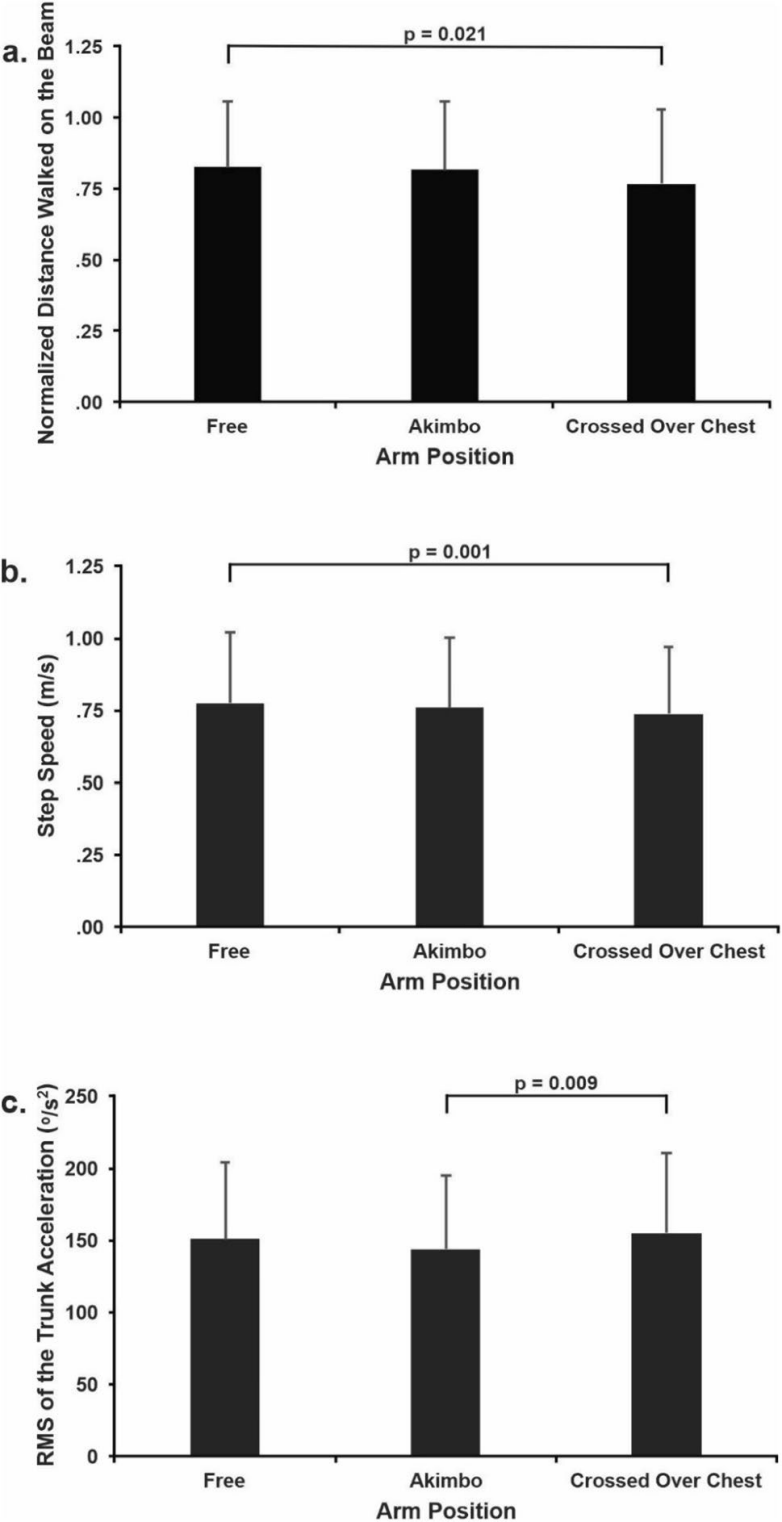
Mean and standard deviation of the arm position main effect for the normalized walking distance on the beam (**a**), step speed (**b**), and RMS of the trunk acceleration (**c**). The horizontal lines indicate pairwise differences with the respective *p*-value.

There was a main effect of arm position for the normalized distance walked on the beams (F_1.625, 30.876_ = 4.784; *p* = 0.021; ${\eta }_{p}^{2}$=0.201), step speed (F_1.893, 35.976_ = 8.775; *p* = 0.001; ${\eta }_{p}^{2}$=0.316), and trunk acceleration RMS (F_1.872, 35.562_ = 5.56; *p* = 0.009; ${\eta }_{p}^{2}$=0.226). Beam distance walked was 7.2% shorter with arms crossed (0.77 ± 0.26) than with arms free (0.83 ± 0.23; d = 0.37, Fig. 3A). When walking with arms crossed (0.74 ± 0.23 m/s), older adults also walked 5.1% slower than with arms free (0.78 ± 0.25 m/s, d = 0.22, Fig. 3B). Surprisingly, trunk acceleration RMS in the akimbo position (144.16 ± 50.44 º/s^2^; d = 0.28) was 7.1% lower than in the arms crossed condition (155.16 ± 55.82 º/s^2^, Fig. 3C). Arm position did not have any effect on RMS of COM displacement or cognitive error (see Table 2 for means and standard deviations for all conditions).

### Cognitive task and arm position interaction

**Figure 4:**
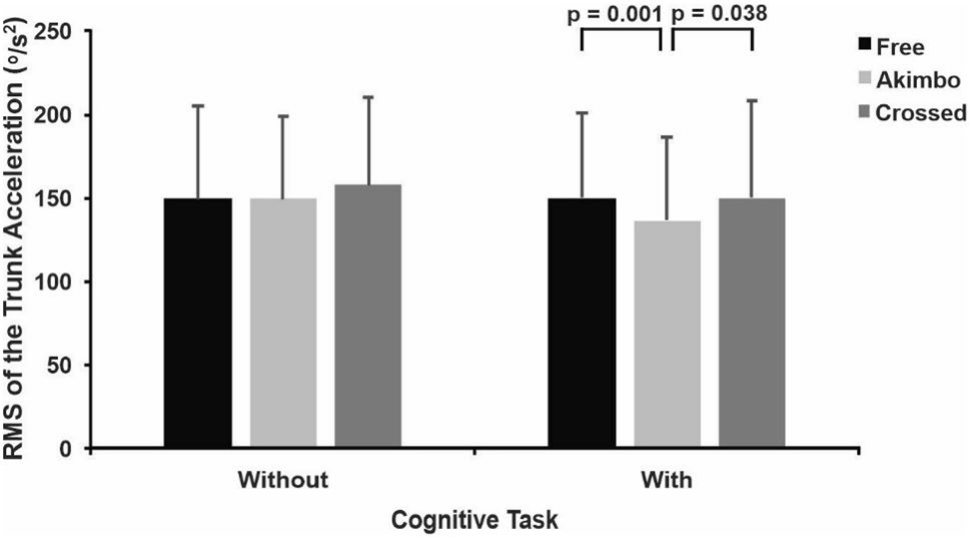
Mean and standard deviation of the interaction between cognitive task and arm position for the RMS of the trunk acceleration. The horizontal lines indicate pairwise differences with the respective *p*-value.

There was an interaction between cognitive task and arm position only for trunk acceleration RMS (F_1.808, 34.349_ = 3.534; *p* = 0.044; ${\eta }_{p}^{2}$=0.157). Trunk acceleration RMS was 9.0% and 9.2% lower in the cognitive task plus arms akimbo condition (137.59 ± 50.16 º/s^2^) compared with the cognitive task plus arms free (151.24 ± 51.15 º/s^2^; *p* = 0.001) and cognitive task plus arms crossed conditions (151.49 ± 58.45 º/s^2^; *p* = 0.038), respectively (Fig. 4). The ANOVA identified no other interaction.

## Discussion

We examined if and how manipulations of beam width, cognitive task, and arm position would affect dynamic balance during beam walking in older adults. Against expectations of interactive effects on dynamic balance, we mainly observed additive effects of beam width, cognitive task, and arm position on beam walking metrics. Only an interaction between cognitive task and arm position was identified for trunk acceleration RMS.

Reducing beam width produced monotonically decreasing beam walking distance (Fig. 1A) and increasing trunk accelerations (Fig. 1C). In contrast, step speed slowed only on the narrowest beam (Fig. 1B), showing beam width did not affect the variables uniformly. The assessment of distance walked and step speed reveals differences in both balance performance and the strategies used to achieve that performance. Beam walking distance tends to increase with foot placement accuracy^3, 10^ and more so in older adults^3, 10^. Indeed, step accuracy increases when one walks slower on the beam^10^. Our current data are consistent with the idea that our older adults slowed beam walking speed when beam width decreased to 6 cm, the narrowest^10^. This strategy is not affected by small differences in beam widths between studies, as the slowing strategy was present for the narrowest beam for widths of 12, 8, and 4 cm^10^ and 10, 8, and 6 cm in the present study. Despite slowing walking speed in an effort to stay on the narrow beam and walk a longer distance, the beam width effect is so robust that it negates reductions in speed and participants still end up walking the shortest distance on the narrowest beam.

In the attempt to walk further and increase dynamic balance, trunk acceleration RMS increased by 24% with beam width decreasing, but the RMS of COM displacement was not affected. Trunk acceleration RMS increases when the walking surface is manipulated^17^ or when walking speed increases^18^. Interestingly, and in contrast with the current results, trunk acceleration RMS in a previous study was similar when walking on a beam and a tape fixed to the floor^19^. Those authors argued that older adults increased trunk stiffness, which can reduce trunk acceleration while walking on a beam. However, trunk stiffness also reduces flexibility to adapt to any eventual oncoming perturbations while walking on the beam. In the present study, considering the lack of difference in the RMS of COM displacement, the increase in trunk acceleration RMS in narrower beams may suggest a different strategy: instead of stiffening the trunk, moving the trunk in an attempt to keep stability and stay longer on the beam, even when the performance is not maintained. Increased trunk movements suggest a need for constant adjustments to try to keep COM within the BoS, maintain dynamic balance, and walk further. However, the desired outcome is not always achieved, as seen with the decrease in distance walked on the beam along beam narrowing. Frequent online adjustments could underlie the changes in trunk acceleration to ensure dynamic balance. Such adjustments are crucial for COM control, as trunk mass corresponds to over 40% of whole-body mass^20^. Other options are not readily available because as the base of support decreases with decreasing beam width, the use of an ankle strategy is limited because it is difficult to produce sufficient ankle torque to restore balance^21^. Therefore, dynamic balance during beam walking is predominantly controlled by adjustments based on a hip strategy, which inevitably involves trunk movements. In sum, measuring distance walked and step speed under such conditions could not only quantify dynamic balance performance but could also provide insights into the underlying control mechanisms. However, the monotonic reductions in beam walking distance, reaching almost 30% in the narrowest beam, also suggest that this trunk strategy has its limits. Indeed, high trunk accelerations are typically associated with poor postural control^19^.

The expectation that a cognitive task affects walking speed is based on the overlapping activation of specific brain areas during walking and cognitive tasks^22^. It is possible that walking speed use different pathways than other changes required to maintain balance. Perhaps this path is linked with the frontal area, which is also responsible for cognitive resources. Motor cortical and white matter properties decline with aging and such reductions are associated with changes in walking kinematics^23–25^. Even in healthy, community-dwelling older adults, cognitive dual-tasking reduces walking speed by 0.19 m/s^26, 27^. In agreement with these neuro-motor-cognitive data, in the present study, the cognitive task reduced step speed by 15% (Fig. 2) compared to the single-task condition. However, no decline was observed in the distance walked on the beam with the cognitive task. In this way, our data partially disagree with a previous study reporting decreases in both beam walking distance and speed while using a similar cognitive task^10^. Even with comparable beam walking distances between cognitive conditions, it appears that our participants prioritized the cognitive task over the motor task, as they walked slower on the beam than without the cognitive task. The reduction in step speed reflects a bottleneck of neural resources between motor and cognitive tasks, known to cause processing delays, which are also associated with age-related changes in brain properties^28^. Prioritizing cognition over walking speed is also supported by a lack of increase in cognitive errors while dual-tasking. A previous study did not find any difference in cognitive performance between walking on a narrow or wide path^29^, which supports our result. It remains to be determined if a difference of 2 cm in beam width and/or diversity in participants’ fitness underlie the differences in beam walking distance in the present and the previous study^10^. In the previous study, participants walked approximately 0.25 m/s slower than our participants; hence they spent much more time on the beam, leading eventually to a loss of balance, step-off, and a shorter beam walking distance^10^. From a clinical perspective, it remains unclear if cognitive dual-tasking while walking on a narrow beam would increase the sensitivity of beam walking distance to detect subtle changes in motor-cognitive dysfunction, especially in highly fit, community-dwelling older adults. However, more fragile populations can be more susceptible to cognitive load changes during a challenging walking task such as beam walking.

Unlike the cognitive task but similar to beam width, arm position strongly affected beam walking distance, step speed, and trunk acceleration RMS but not RMS of COM displacement. For the most part, walking on the beam with arms crossed was the most challenging of the three arm positions, decreasing walking distance by 7.2% (d = 0.37) and step speed by 5.1% (d = 0.22), as shown in Figs. 3A and B, respectively. These data agree with previous reports of reduced beam walking performance and speed when walking with arms restricted^11, 30^. With the arms free, participants perform high acceleration and high amplitude arm movements to maintain balance on the beam, which also moves the trunk to keep balance. In contrast, walking with the arms crossed, the arms and the trunk form one anatomical structure, decreasing the rotational inertia^30^, and making it a single strategic element of balance control. Both strategies affect trunk acceleration variability. In addition to the trunk, hip movements also contribute to trunk and balance control, as the narrow BoS limits contributions from the ankle to balance control. One would expect that trunk acceleration RMS would differ between conditions of walking with and without arms free. We suspect that the slower walking speed while walking with arms crossed versus arms free is related to no differences in trunk acceleration RMS, as walking speed is associated with trunk acceleration^19^. Our data expand the emerging picture that walking on a narrow beam with arms crossed, a truly challenging balance task, necessitates the engagement of trunk strategy^5^. Nonetheless, arm movements are not considered in either balance strategy^5^, calling for future studies to verify the role of upper body movements in balance control during beam walking.

While akimbo position did not affect walking distance, it decreased trunk acceleration variability, implying an altered walking strategy to maintain balance. We propose that the akimbo position could increase rotational inertia, allowing individuals to have a slightly better balance control by only moving the elbows back and forth, minimizing the arm actions in relation to the trunk, and reducing its influence on trunk acceleration. Taken together, manipulating arm position during beam walking could increase the sensitivity of this test to detect subtle balance dysfunctions. Future investigations should also manipulate arm position during a challenging walking task, e.g., beam walking, in the clinical population to assess dynamic balance impairments more accurately in disease.

The only interaction we identified was between cognitive task and arm position for trunk acceleration RMS, showing that the akimbo position reduced trunk acceleration only in the dual-task condition. The interactive effect highlighted the influence of akimbo position in decreasing the trunk acceleration RMS, being different not only from crossed, but also from free arms in the presence of the CT. A previous study suggested that upper body movements incrementally complement hip and ankle strategies in balance regulation in more difficult conditions^5^. Hence, when the cognitive task is added, the difficulty level increases suggesting the akimbo position was the only position capable of increasing trunk control, which was needed in the dual-task condition for participants to focus more on the concomitant cognitive task.

Against the hypothesis, we observed no interaction effects between beam width, arm position, and cognitive task during beam walking. In the present study, task difficulty of a particular condition was the simple sum of the complexity of the individual manipulations. It is possible that each perturbation was unique, affecting one element of beam walking independently and minimizing other perturbation effects. However, a lack of interaction among the three perturbations is unexpected in light of previous work^10^, which reported increasingly poorer beam walking performance with a combination of perturbations such as beam width and cognitive task. In the present study, the effects of the manipulations are summed to influence beam walking, but they do not interact with each other.

We did not perform this study without limitations. Using a sample of older adults recruited by convenience resulted in participants with a high level of physical activity and unimpaired functional balance, limiting the generalization of our findings to this population. In addition, it was already seen that anxiety can increase the allocation of attention to locomotor control and deteriorate motor and cognitive tasks^31, 32^. In this way, not assessing the fear of falling of the participants left room for questioning if the performance was affected by some level of anxiety during beam walking. Besides that, the fixed order of trial blocks was designed to avoid fatigue and demotivation, but could also promote carry-over effects between conditions, minimizing the impact of the manipulations. Analyzing only the successful steps can also hide some additive, but mainly interactive effects of beam width, cognitive task, and arm position. There is no space for the factors to interact and create even more difficult conditions without making the participant lose balance. Also, not analyzing arm kinematic data limited our argument about its influence on trunk acceleration behavior. In this way, further studies with randomized conditions, more fragile older adults, and analysis of arm influence and the steps off are required to expand our findings.

In conclusion, distance walked indicated that beam width and arm position, but less so cognitive task, affected dynamic balance, implying a great potential to become a sensitive test of subtle impairments. Stability measurements suggested effective trunk adjustments to control COM position and keep dynamic balance during the task.

## Methods

### Participants

We used a convenience sample recruited from exercise programs offered to older adults. Inclusion criteria were age ≥ 65 years, a score of > 7 on the Short Physical Performance Battery (SPPB)^33^, and the ability to understand instructions and walk without assistance. Exclusion criteria were visual, musculoskeletal, cognitive, vestibular, or somatosensory impairments that could affect dynamic balance. We used the International Physical Activity Questionnaire (IPAQ) to determine physical activity level^34^, Mini-Mental State Examination for cognitive evaluation^35^, Mini-BEST Test to estimate functional balance^36^, Trail Making Test—Parts A and B to evaluate attention and executive function, respectively^37^, and Digit Symbol Substitution Test to assess the processing speed and intelligence fluidity^38^. The procedures of this study were performed in accordance with the Declaration of Helsinki and were approved by The Research Ethics Committee of the School of Physical Education and Sport of Ribeirão Preto (CAAE: 92534818.5.0000.5659). All participants signed the informed consent document before testing started.

### Procedures and data collection

We placed 39 passive reflective markers over specific anatomical landmarks according to the Plug-in Gait Full Body model to reconstruct fifteen segments and estimate the COM position. We used a 10-camera motion capture system (Vicon, Oxford, UK) to track the three-dimensional trajectories of those markers at 200 Hz.

**Figure 5:**
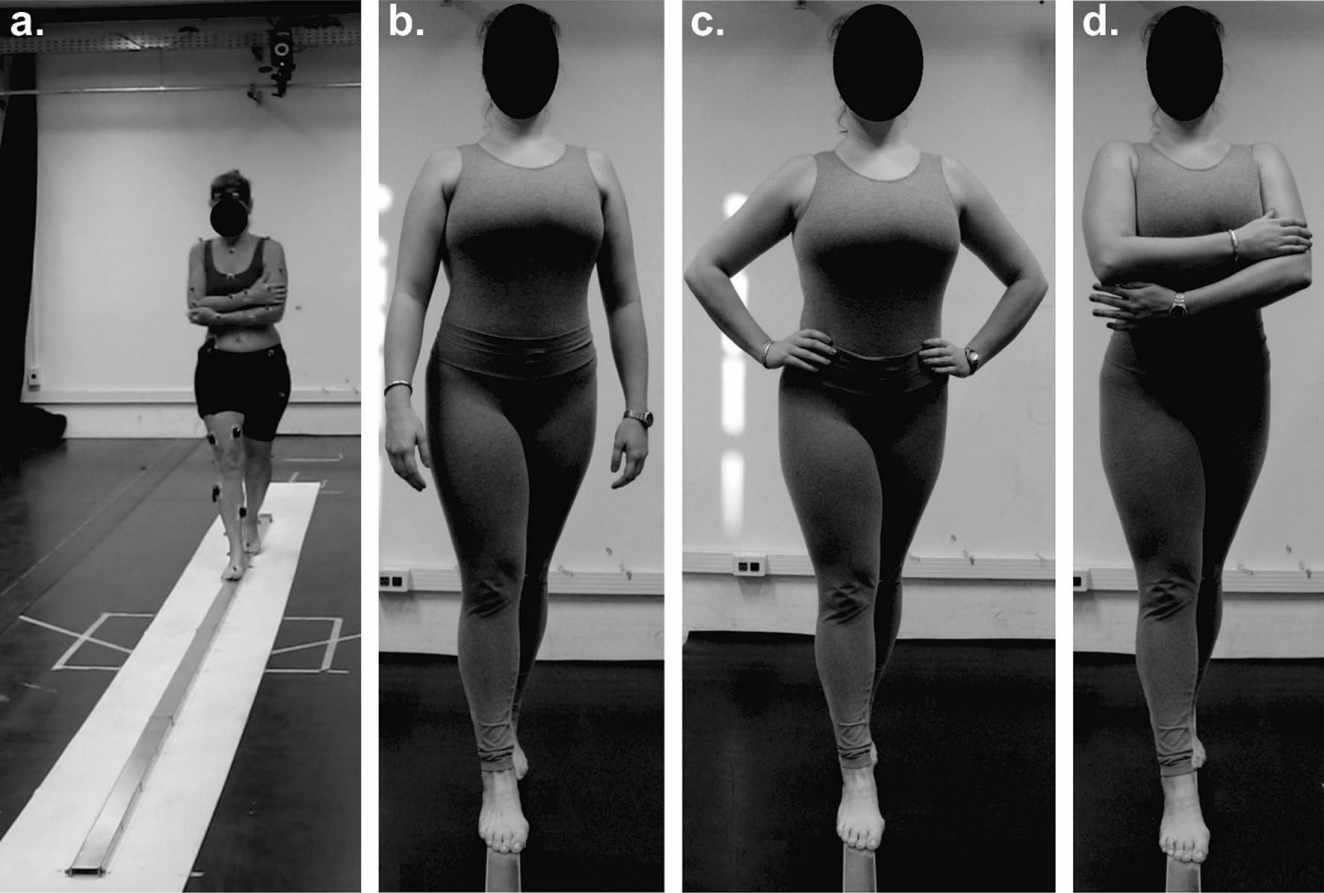
Representation of the experimental setup (**a**) and the three arm positions: free arms (**b**), akimbo (**c**), and crossed arms (**d**).

Participants walked at their preferred speed on aluminum beams, following the instruction “Walk on the beam safely without stepping off”. No orientation about task prioritization was given in the dual-task condition. The beams were 4 m long, 2 cm high, and had three different widths: 10, 8, and 6 cm. Each beam width composed a block of trials with three arm positions randomized within it. The arm positions were free arms, akimbo, and crossed over chest (Fig. 5). Each arm position was performed three times, totalizing nine trials in each beam width block. Participants performed each block without and with a concurrent cognitive task, which consisted of a sequential subtraction by 3, starting at a random number between 300 and 900. Participants verbalized the results of each subtraction while walking. The blocks were performed in order of increasing difficulty, i.e., 10-, 8- and 6-cm beams, without the cognitive task, and then we repeated the same order with the cognitive task.

### Data analysis

Only successful trials were analyzed during which participants maintained arm positions (akimbo, chest-crossed) and kept their feet on the beam. The first and last steps from each trial were excluded from the analyses to minimize acceleration and deceleration effects.

Marker coordinates were filtered with a low-pass, 4th-order Butterworth digital filter using a cut-off frequency of 6 Hz. Marker coordinates were used to calculate step speed, COM displacement, and trunk angular acceleration. Step speed was computed as the ratio between step length (difference between the anterior–posterior coordinates of successive heel contacts) and step duration (time difference between successive heel contacts). Whole-body COM position was estimated with a 15-segment model^39^. Trunk angle was computed as the absolute angle between the trunk, i.e., the segment formed by the midpoints between Clavicle and C7, Sternum and T10, and Clavicle and Sternum, and the laboratory coordinate system. Trunk angular acceleration was calculated by taking the second derivative of the trunk angle (i.e., lateral inclination). Trunk acceleration was used to assess trunk control during dynamic balance, including beam walking^19, 40, 41^. The root mean square (RMS) measures dispersion within the time series^41^, and higher values are associated with poorer balance^19^. Thus, the RMS for each 1-s window, and afterward, the mean value of these windows was calculated for COM displacement and trunk acceleration in the frontal plane.

We considered all successful steps to measure walking distance, including the first and last ones. Normalized distance walked was calculated as the quotient of the sum of the distance traveled over the three trials and the maximum possible distance for each condition (i.e., 12 m)^6^. A normalized distance of 1.0 indicates perfect performance (i.e., no step-offs). Distance indicators were placed at 0.25 m increments along the beams to help ascertain the step-off location to the nearest cm based on views from two video cameras. These same cameras recorded the cognitive performance for posterior analysis.

For cognitive error, we evaluated the ratio between the number of incorrect subtraction responses and the number of the total responses (i.e., cognitive error), normalized by the walking distance on each trial.

### Statistical analyses

We performed 3 beam widths × 3 arm positions × 2 cognitive task conditions 3-way ANOVA with all factors as repeated measures for the normalized distance walked, step speed, RMS of COM displacement, and trunk acceleration RMS. A two-way ANOVA (3 beam widths × 3 arm positions) was run for the cognitive error with repeated measures in all factors. Post-hoc tests with Bonferroni corrections were used for main and interaction effects. Greenhouse–Geisser corrections were used to adjust p-values when the assumption of sphericity was not met, as demonstrated by a significant Mauchly’s Test of Sphericity (*p* ≤ 0.05). The effect size was estimated using partial eta squared (${\eta }_{p}^{2}$) for ANOVA and Cohen’s d for pairwise comparisons (small: 0.2; medium: 0.5; large: 0.8). The level of significance was set at *p* ≤ 0.05.

## Supplementary Information

Supplementary Information.

## Data Availability

All data generated in this study are included as a Supplementary Information file.
